# The increasing role of breast-conserving treatment in patients with BRCA-related breast cancer: an appraisal of recent literature

**DOI:** 10.3389/fonc.2026.1861028

**Published:** 2026-07-29

**Authors:** Alessandro Fancellu, Giuliana Giuliani, Silvia Mulas, Anna Maria Contini, Maria Laura Ariu, Claudia Piredda, Valeria Sanna

**Affiliations:** 1 Breast Unit, Azienda Ospedaliero-Universitaria di Sassari, Sassari, Italy; 2 Dept. of Medical Oncology, Azienda Ospedaliero-Universitaria di Sassari, Sassari, Italy

**Keywords:** BRCA-associated breast cancer, breast cancer recurrence, breast conserving therapy, mastectomy, oncologic outcomes

## Abstract

Breast-conserving therapy (BCT) in BRCA-associated breast cancer remains a subject of ongoing debate, primarily due to concerns regarding local recurrence and the development of second primary tumors. This review provides a critical appraisal of the current evidence comparing BCT and mastectomy, integrating oncologic outcomes with biological insights and patient-centered perspectives. Evidence from cohort studies and meta-analyses indicates that, although BCT is associated with a higher incidence of ipsilateral breast events, this does not translate into inferior long-term survival. This apparent divergence may reflect the distinct biological profile of BRCA-associated tumors, characterized by impaired DNA repair mechanisms and increased responsiveness to systemic treatments, which may enhance therapeutic efficacy, particularly in the neoadjuvant setting. Recent advances in systemic therapies, including PARP inhibitors, along with improvements in radiotherapy and imaging, have broadened the clinical applicability of breast-conserving strategies. Furthermore, considerations related to quality of life and patient preferences reinforce the importance of individualized surgical planning. In this context, BCT can be considered a valid and oncologically sound option for selected BRCA mutation carriers, supporting a tailored multidisciplinary approach to treatment decision-making.

## 1 Introduction

Breast cancer associated with germline mutations in BRCA1 and BRCA2 represents a distinct clinical entity characterized by a significantly increased risk of developing breast and ovarian cancer, as well as other malignancies. Women carrying pathogenic BRCA1 or BRCA2 variants have a lifetime breast cancer risk of approximately 60–70%, although penetrance varies according to population characteristics and mutation-specific factors. Breast cancers arising in BRCA mutation carriers account for approximately 5–10% of all cases, with higher prevalence observed in younger patients and in those with a strong family history of cancer (1–4).

BRCA-associated breast cancers also exhibit distinct biological and clinical features, including a higher prevalence of triple-negative phenotype in BRCA1 carriers and increased genomic instability, which may influence treatment response and long-term outcomes. Furthermore, these patients face a significantly increased risk of ipsilateral tumor recurrence (IBTR) and contralateral breast cancer (CBC), which has historically influenced surgical decision-making.

Surgical management in patients with BRCA-associated breast cancer has favored mastectomy, largely driven by concerns regarding local recurrence, multicentric disease, and the elevated risk of CBC (3, 5, 6). This approach has also been reinforced by the perception of BRCA-associated tumors as biologically aggressive and prone to recurrence. However, accumulating evidence over the past decade suggests that breast-conserving therapy (BCT) may provide oncological outcomes that are comparable to those of mastectomy in appropriately selected patients. Advances in neoadjuvant chemotherapy, PARP inhibitors, imaging, and patient selection have further expanded the feasibility and safety of breast-conserving approaches in this high-risk population (7, 8). This evolving body of evidence has challenged the long-standing paradigm of mastectomy as the mandatory surgical approach in patients with BRCA-associated breast cancer, opening the way to more individualized, risk-adapted surgical strategies.

The present review aims to critically evaluate the current evidence on the role of BCT in BRCA-associated breast cancer, with a focus on oncological outcomes, patient selection, and ongoing controversies, while outlining future perspectives in this rapidly evolving field.

## 2 Methods

A comprehensive literature search was performed using the PubMed database to identify relevant studies evaluating surgical management and oncological outcomes in patients with BRCA-associated breast cancer. The search strategy included combinations of the following terms: “BRCA1”, “BRCA2”, “breast cancer”, “hereditary”, “breast-conserving therapy”, “breast-conserving surgery”, “mastectomy”, and “outcomes”.

Eligible studies included original research articles, systematic reviews, and meta-analyses reporting data on local recurrence, disease-free survival, and overall survival. Reference lists of retrieved articles were also screened to identify additional relevant publications. Study selection was based on relevance, methodological quality, and recency of evidence, with a focus on comparative studies between BCT and mastectomy in BRCA mutation carriers. Non-English articles and studies lacking clinically meaningful outcome data were excluded.

**Figure 1:**
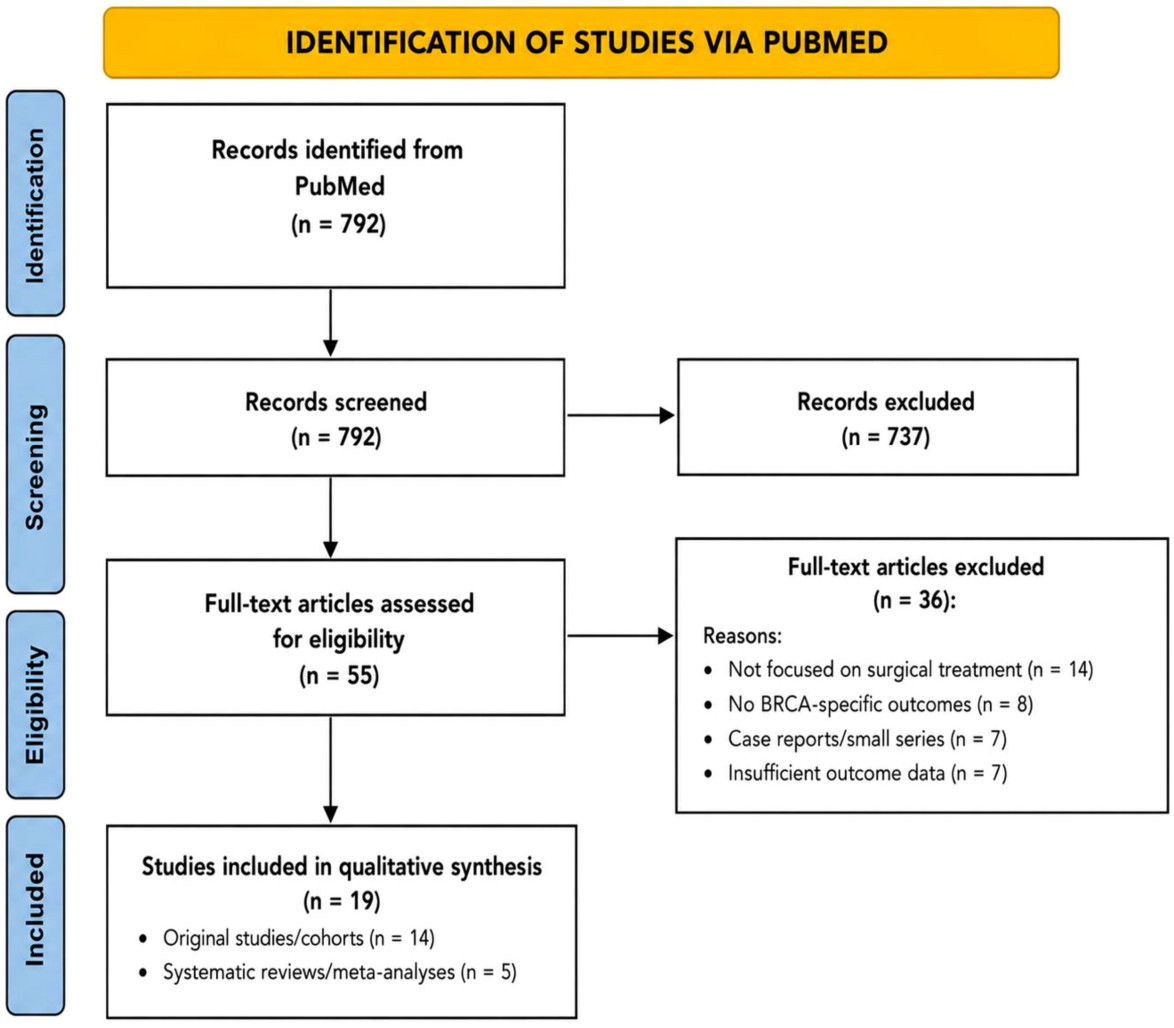
PRISMA-style flowchart of literature search and study selection.

A PRISMA-style flow diagram has now been included as **Figure 1** and is referenced in the Methods section. The diagram details the identification of 792 records through PubMed searching, the screening process, eligibility assessment, and the final inclusion of 19 studies (14 original studies and 5 systematic reviews/meta-analyses).

## 3 Biological and clinical features of BRCA-associated breast cancer

BRCA1 and BRCA2 mutations impair homologous recombination repair, a key pathway for the accurate repair of double-strand DNA breaks, leading to genomic instability and accumulation of DNA damage (1, 2). This deficiency contributes to the development of tumors with distinct biological and pathological features, including higher histological grade, elevated proliferation indices, and marked genomic instability. In particular, BRCA1-associated breast cancers are more frequently characterized by a triple-negative phenotype, which is typically associated with a more aggressive clinical course and limited targeted treatment options (2). In contrast, BRCA2-associated breast cancers more commonly exhibit hormone receptor-positive (estrogen receptor-positive) phenotypes and share several clinicopathological features with sporadic luminal-type breast cancers, although they retain defects in DNA repair mechanisms that may influence therapeutic sensitivity (1). Compared with BRCA1 carriers, BRCA2 mutation carriers tend to present with lower-grade tumors and a less frequent triple-negative phenotype, while maintaining a substantial lifetime risk of breast cancer.BRCA mutation carriers typically develop breast cancer at a younger age compared with the general population. The median age at diagnosis is often reported in the fourth to fifth decade of life, particularly among BRCA1 carriers, whereas BRCA2-associated cancers may present slightly later but still significantly earlier than sporadic cases (3). Early age of onset, combined with the elevated lifetime risk of both IBTR and CBC, has important implications for surveillance strategies and surgical decision-making.

Despite these aggressive or high-risk features, BRCA-associated tumors demonstrate a paradoxical increased sensitivity to DNA-damaging agents. This unique biological behavior has important clinical implications, especially in the neoadjuvant setting. Several studies have reported higher rates of pathological complete response in BRCA mutation carriers compared with non-carriers, particularly in triple-negative breast cancer, thereby increasing the likelihood of tumor downstaging and eligibility for breast-conserving approaches (2, 9–11). Furthermore, improved systemic disease control achieved with modern chemotherapy regimens and targeted therapies may reduce the relative impact of local recurrence on long-term outcomes, supporting a more conservative surgical strategy in appropriately selected patients.

## 4 Historical perspective

Traditionally, mastectomy has been considered the standard surgical approach for patients with BRCA-associated breast cancer, primarily due to concerns regarding higher rates of IBTR, multicentric disease, and the increased risk of second primary and CBC (12–14). These concerns were further reinforced by the perception of BRCA-associated tumors as biologically aggressive, particularly in BRCA1 mutation carriers, who more frequently develop high-grade, triple-negative breast cancers.

Early retrospective studies suggested higher local recurrence rates following BCT compared with sporadic breast cancer, leading many clinicians to favor mastectomy as a more definitive oncological approach (12). Additionally, the elevated risk of subsequent primary tumors in the residual breast tissue contributed to concerns regarding the long-term safety of conservative strategies (14).

However, it is important to recognize that much of the historical evidence supporting mastectomy was generated in an era preceding significant advances in systemic therapy and radiotherapy. Earlier studies often lacked the use of contemporary chemotherapy regimens, including taxane- and platinum-based therapies, as well as modern targeted treatments. Furthermore, radiotherapy techniques were less refined, and imaging modalities were less sensitive, potentially contributing to higher observed rates of local failure (13, 15, 16).

Another limitation of early studies was the inadequate consideration of tumor biology and genetic heterogeneity. Differences between BRCA1 and BRCA2-associated tumors, as well as variability in treatment strategies, were not consistently accounted for, limiting the applicability of these findings to current clinical practice.

As a result, in recent years, the historical paradigm favoring mastectomy as the default surgical strategy has been increasingly questioned, supporting a shift toward more individualized, biology-driven surgical decision-making.

## 5 Oncologic outcomes of BCT in patients with BRCA-associated breast cancer

Observational studies have investigated the oncologic safety of BCT in patients with BRCA-associated breast cancer, initially yielding heterogeneous results but progressively converging toward more consistent conclusions. The **Table 1** reports the results of retrospective studies in which BCT was used in BRCA mutations carriers diagnosed with breast cancer.

**Table 1:** Studies investigating the oncologic outcomes of BCT in patients with BRCA-associated breast cancer.

Author (year)	Country	Type of study	Patients’ characteristics	No of patients/surgical treatment	Length of follow-up (median)	Oncologic outcomes	Main conclusions
Haffty (12)	United States	Retrospective cohort study	Patients ≤ 42 years receiving BCT for breast cancer	22 patients with BRCA mutation 105 patients with sporadic disease	Up to 12 years follow-up	Patients with BRCA-associated breast cancer had significantly higher rates of ipsilateral (P 0.007) and contralateral events (P 0·001) than the sporadic group	Patients with germline mutations in *BRCA1* or *BRCA2* have a high risk of developing late ipsilateral and contralateral second primary tumours.
Robson (32)	United States	Retrospective cohort study	BRCA-associated breast cancer	87 underwent BCT	6.3 years^*^	5-year and 10-year probabilities of metachronous IBC were 11.2% and 13.6%, respectively. 5-year and 10-year probabilities of CBC after were 11.9% and 37.6%.	Women with *BRCA*-associated breast carcinoma who undergo BCT appear to have risks of metachronous IBC similar to those reported for young women without known mutations. Significant risks of CBC and possibly late MIBC may prompt the serious consideration of bilateral mastectomy as a preventive measure.
Garcia-Etienne (6)	Italy	Matched-cohort study	BRCA-associated breast cancer	54 BRCA mutated 162 sporadic breast cancer	4 years	Ten-year cumulative incidence of IBTR was 27% for mutation carriers and 4% for sporadic controls (P = 0.03). Ten-year cumulative incidence of CBC was 25% for mutation carriers and 1% for sporadic controls (P = 0.03).	Increased risk of IBTR and CBC should be discussed with carriers interested in breast conservation as well as when choosing risk-reducing strategies.
Pierce (13)	United States	Multicenter observational cohort study	Patients with BRCA1/2 mutations diagnosed with breast cancer	302 patients underwent BCT 353 patients underwent Mastectomy	8.2 years (BCT) 8.9 years (Mastectomy)	BCSS and OS were not different between BCT and Mastectomy (P 0.85 and P 0.73, respectively). Local failure was higher after BCT (p<0.0001). CBC was higher in BRCA/1 than in BRCA/2 carriers	Patients with BRCA1/2 associated breast cancer have similar survivals whether treated with mastectomy or BCT. After BCT, higher risk of a second in breast event (reduced with chemotherapy). High risk of CBC (>40%), independent of surgical approach.
Metcalfe (14)	Canada, United States	retrospective multicenter cohort study	Women with stage I/II breast cancer with BRCA1 or BRCA2 mutation identified in the family	396 patients underwent BCS^**^	10.5 years*	The 5-year actuarial risk of ipsilateral breast cancer was 5.8% and the 10-year risk was 12.9%.	BCS in BRCA mutation carriers is associated with a moderate long-term risk of ipsilateral breast cancer (13% at 10 years), which can be significantly reduced by radiotherapy, chemotherapy, and oophorectomy.
Nilsson (17)	Sweden	retrospective cohort study	Women with BRCA1/2 associated breast cancer	45 patients underwent BCT 118 patients underwent Mastectomy	Long-term follow-up (up to 15 years)	The cumulative risk of LRR was higher in the BCT group (P 0.003).	No differences between BCT and Mastectomy for OS, breast cancer death, or distant recurrence. BRCA1/2 mutation carriers treated with BCT have a high risk of LRR.
Ye (22)	China	Retrospective cohort study	Patients receiving BCS for breast cancer	63 patients with BRCA 1/2 mutation	5 years (BRCA-carriers) - 5.8 years (non-carriers)	OS, DFS and IBTR were not different between BRCA-carries and non carriers (P .17, P 0.42, P 0.34 respectively). CBC higher in BRCA carriers (P < .001).	BRCA mutation carriers have higher risk of CBC
van de Broek (24)	The Netherlands	retrospective multicenter cohort study	women with invasive breast cancer diagnosed <50 years	261 with BRCA 1/2 mutation, of whom 91 BRCA1carriers underwent BCT	14.8 years	Similar OS after BCT between BRCA1 carriers and no-carriers (P .41). LRR rates after BCT did not differ between BRCA1 carriers and no-carriers (10-year risk 7.3% vs 7.9%).	BCT is a safe local treatment option to offer to BRCA1 mutation carriers with invasive breast cancer.
Ye (48)	China	Retrospective cohort study	Early-onset triple-negative breast cancer	67 patients BRCA mutated, of whom 27 underwent BCS^¶^ 40 underwent Mastectomy	~ 5 years	Clinical outcomes comparable between BCT and Mastectomy in patients with BRCA mutations: OS (p 0.30) and DFS (p 0.22)	BCT could be a safe alternative surgical option for early-onset TNBC patients with BRCA mutations. Early onset TNBC patients with BRCA mutations are prone to better OS and BCSS compared to BRCA-wild.
Gentile (18)	Italy	Retrospective cohort study	BRCA-associated breast cancer	69 underwent BCT 55 underwent Mastectomy 72 also underwent BSO	~ 4,3 years	No difference in DFS, DDFS, and OS between BCT or Mastectomy (p 0.39, p 0.27, p 0.265, respectively). Patients treated with BSO had significantly better DDFS and OS compared to ovarian conservation (p 0.033,p 0.040, respectively).	BRCA-mutation carriers treated with BCT present similar oncological outcomes compared to mastectomy. Ovarian preservation was associated with worse survival outcomes. Young BRCA-mutated patients with small BCs may not need up-front mastectomy.
Shubeck (20)	United States	Retrospective cohort study	BRCA-associated breast cancer	395 with 424 cancers, of whom 99 underwent BCS^±^ and 325 underwent Mastectomy	7.9 years	LRR (p 0.4), BCSS (p 0.6), and OS (p 0.7) rates did not differ between groups. 10-year estimated CBC risk of 14% among unilateral breast surgery patients (p<0.001).	Data support breast conservation as an option for BRCA mutation carriers willing to continue high-risk screening.
Wanis (23)	United States	Retrospective cohort study	BRCA 1/2-associated breast cancer	172 underwent BCT	11.8 years	10-year OS was 88.5%, DDFS was 87.0%, IBC event risk was 12.2%, CBC was 21.3%	Although women with breast cancer and pathogenic *BRCA1/2* variants treated with BCS had above-average risks of IBC and CBC events, most did not have another cancer event and remained bilateral mastectomy free.
Mahiou (21)	France	retrospective cohort study	Women with BRCA 1/2 associated breast cancer	69 underwent BCS^#^	7.7 years	no difference in OS (P .25), no difference in RFS (P .22) between breast conservation and Mastectomy	Conservative surgery, when possible, appears to be a feasible option over total mastectomy, with no difference in OS in patients with BRCA 1/2 associated invasive breast cancer.
Lee (19)	South Korea	Retrospective multicenter cohort study	BRCA1-2 associated breast cancer receiving BCT or Mastectomy	367 underwent BCT 186 underwent Mastectomy	8.3 years	No significant difference in oncologic outcomes between BCT and mastectomy: LLRFS (p 0.83), DRFS (p 0.18), RFS (p 0.14), OS (p 0.87)	BCT with close surveillance can be considered a viable treatment option for BRCA1 or BRCA2 pathogenic variant carriers.

BCT, Breast conserving Therapy; MIBC metachronous ipsilateral breast carcinoma; CBC, contralateral breast cancer; IBTR ipsilateral-breast tumor recurrence; BCSS, breast cancer specific survival; OS overall survival; BCS, breast conserving surgery; DFS, disease-free survival; TNBC, triple negative breast cancer; DDFS distant disease-free survival; LRR loco-regional recurrence; IBC, ipsilateral breast cancer; RFS, recurrence-free survival; LRRFS Locoregional Recurrence-Free Survival; DRFS, Distant Recurrence-Free Survival.

^*^ mean value.

^**^ 346 patients (87.4%) received adjuvant radiotherapy.

^¶^ 22 patients (81.5) received adjuvant radiotherapy.

^±^ 64 patients (64.6%) received adjuvant radiotherapy.

^#^ 68 patients (98.5%) received adjuvant radiotherapy.

Early studies reported a significantly increased risk of IBTR and CBC among BRCA mutation carriers undergoing BCT compared with sporadic controls (6, 12). In particular, Haffty et al. demonstrated higher rates of both ipsilateral and contralateral events over long-term follow-up, suggesting a predisposition to second primary tumors rather than true local recurrences (12). Some larger and longer-term cohort studies have shown that BCT is associated with a higher risk of local recurrence compared with mastectomy; however, this did not translate into differences in overall survival (OS) or breast cancer–specific survival (BCSS) (13, 17). Notably, Pierce et al. reported a significantly increased local failure rate after BCT (p < 0.0001), while survival outcomes remained comparable between treatment groups, supporting the hypothesis that many in-breast events represent new primary cancers (13).

More recent evidence has further mitigated concerns regarding the oncologic safety of BCT in BRCA mutation carriers, despite longstanding concerns about ipsilateral breast events. Several retrospective and multicenter studies have demonstrated no significant differences between BCT and mastectomy in terms of OS, disease-free survival (DFS), or distant recurrence (18–21). These findings suggest that, when combined with appropriate adjuvant therapies and close surveillance, BCT represents a safe and effective treatment option in selected BRCA-mutated patients (18, 20). A consistently reported finding across studies is the increased risk of CBC in BRCA mutation carriers, regardless of the type of initial surgery (20, 22, 23). This highlights the importance of individualized risk-reduction strategies and intensive follow-up rather than the sole choice of surgical approach. Studies focusing exclusively on patients treated with BCT have reported a moderate long-term risk of ipsilateral recurrence, generally ranging from 10% to 15% at 10 years, which can be significantly reduced by adjuvant radiotherapy, chemotherapy, and oophorectomy as reported in specific cohorts (14). Consistently, more recent analyses have shown that local recurrence rates after BCT particularly in BRCA1 carriers may be comparable to those observed in non-carriers (24).

In the broader context of early breast cancer, recent literature has also suggested a potential survival advantage associated with breast-conserving approaches compared with mastectomy, likely reflecting a combination of treatment selection, radiotherapy effects, and tumor biology (9, 25).

The available evidence from systematic reviews and meta-analyses consistently supports the oncologic safety of BCT in BRCA mutation carriers, although with some important nuances, primarily regarding the risk of ipsilateral events following BCT (**Table 2**). The earliest meta-analysis by Valachis et al. demonstrated no significant overall increase in IBTR compared with non-carriers (RR 1.45, 95% CI 0.98–2.14), although a significantly higher risk emerged in studies with longer follow-up (≥7 years), suggesting a time-dependent effect likely related to new primary tumors rather than true recurrences (26). This finding has been consistently confirmed by subsequent analyses. More recent meta-analyses directly comparing BCT and mastectomy have consistently shown that, although BCT is associated with a significantly higher risk of locoregional recurrence, survival outcomes remain equivalent. In particular, Davey et al. reported no differences in OS, DFS, or BCSS between the two surgical approaches, and no significant difference in contralateral breast cancer risk, despite a significantly increased risk of local recurrence with BCT (27). Similarly, Wang et al. confirmed that BCT significantly increases the risk of local recurrence but has no impact on no significant impact on survival outcomes (28). Co et al., in a systematic review, also demonstrated comparable OS at 5, 10, and 15 years between BCT and mastectomy, despite higher IBTR rates in the BCT group (8).

**Table 2:** Meta-analyses/systematic review reporting on the use of BCT vs mastectomy in women with BRCA associated BC.

Author (year)	No. of studies included	Follow-up duration	OS (BCT vs mastectomy)	CBC risk	IBTR risk	Main conclusions
Valachis (26)	23	Variable (≥7 yrs subgroup)	Not clearly reported	CBCs were significantly greaterin BRCA-mutation carriers versus controls (RR 3.56).	No difference in IBTR between BRCA-mutation carriers and controls (RR 1.45). Higher risk for IBTR in BRCA-mutation carriers in studies with a median follow-up ≥7 years (RR 1.51). Adjuvant chemotherapy and oophorectomy associated with lower risk for IBTR in BRCA-mutation carriers.	Use of BCT in BRCA-mutation carriers can be considered a reasonable option since BCT does not significantly increase IBTR overall, but higher risk emerges with longer follow-up.
Co (8)*	18	Up to 15 yrs	OS at 5, 10, and 15 years comparable between BCS and mastectomy (88.7%, 89.0% and 83.6% with BCS and 83%, 86.0%, and 83.2% with mastectomy, respectively).	Not consistently reported across studies	IBTR rates at 5, 10, and 15 years were higher in the BCS group (8.2%, 15.5%, and 23%, respectively) than in the mastectomy group (3.4%, 4.9%, and 6.4%, respectively).	BCS is associated with a greater rate of IBTR in BRCA mutation carriers. However, it was not associated with adverse short- and long-term survival outcomes.
Davey (27)	23	~8 yrs	Risk of death equivalent for combined BCT and mastectomy (HR:1.10).	No significant difference between BCT and mastectomy (HR:1.51)	Increased risk of LRR in patients treated with BCT (HR:4.54)..	Survival outcomes are comparable between BCT and mastectomy in BRCA-mutation carriers. Risk of LRR is increased after BCT.
Wang (28)	4 (5 cohorts)	5–15 yrs	No difference between BCT and mastectomy (*p* = 0.925)	Not analyzed. However, patients who received mastectomy tended to have prophylactic contralateral mastectomy (*p* < 0.001)	BCT had higher risk for local recurrence than mastectomy (*p* < 0.001).	BCT increases LLR risk, but did not significantly impacts patient survival in terms of DFS, MFS, BCSS and OS.
Nara (29)	13	Variable (≥7–10 yrs)	No difference after BCT between BRCA mutation carriers and sporadic breast cancer patients	Not analyzed	Increased risk of IBTR in BRCA mutation carriers undergoing BCT compared with sporadic breast cancer patients (p<0.001)	BRCA-mutation carriers after BCT have a higher risk of IBTR compared to sporadic breast cancer patients.

*Systematic review.

IBTR, Ipsilateral breast recurrence; CBC Contralateral breast cancer; BCS Breast conserving surgery, OS Overall survival; BCT breast conserving therapy; DFS, Disease-free survival; BCSS, Breast cancer specific survival; metastasis-free survival; LRR, loco-regional recurrence; MFS, metastasis-free survival.

Importantly, no meta-analysis or systematic review has demonstrated a detrimental effect of BCT on OS (29). This dissociation between higher local recurrence and unchanged survival strongly supports the hypothesis that many ipsilateral events in BRCA mutation carriers represent new primary tumors rather than true recurrences. Overall, the current body of evidence from meta-analyses indicates that, although BCT in BRCA mutation carriers is associated with a higher risk of local breast events—particularly over long-term follow-up—it does not adversely affect survival outcomes. Therefore, BCT can be considered a safe and acceptable alternative to mastectomy in selected patients, provided that appropriate counseling, adjuvant treatment, and long-term surveillance are ensured.

## 7 Systemic therapies and radiotherapy

BRCA1/2-mutated breast cancers display distinct biological and therapeutic characteristics compared with non-carriers, largely driven by defects in homologous recombination DNA repair. As a consequence, BRCA-associated tumors are generally more sensitive to DNA-damaging agents, including platinum-based chemotherapy and anthracyclines, which induce double-strand DNA breaks that cannot be effectively repaired in BRCA-deficient cells (30, 31). This enhanced chemosensitivity is particularly evident in BRCA1-mutated and triple-negative breast cancers, where higher response rates to neoadjuvant chemotherapy have been reported compared with sporadic tumors. In addition, systemic therapies appear to play a crucial role in reducing both true recurrences and new primary tumors, which are known to contribute substantially to ipsilateral breast events in BRCA carriers (13, 14). A major advancement in the management of BRCA-mutated breast cancer is the introduction of PARP inhibitors, which exploit the concept of synthetic lethality in homologous recombination-deficient tumors. Agents such as olaparib and talazoparib have demonstrated significant clinical benefit in both early and metastatic settings, further differentiating the therapeutic landscape of BRCA-associated disease from that of non-mutated breast cancer (31, 32).

Endocrine-related interventions also exhibit unique implications in this population. Risk-reducing salpingo-oophorectomy has been associated with a decrease in breast cancer development, particularly in BRCA1 carriers, although the magnitude of its effect may be influenced by age and concomitant systemic therapies. Tamoxifen, on the other hand, appears to reduce the risk of contralateral breast cancer rather than ipsilateral recurrence, with its benefit largely confined to hormone receptor-positive disease (4, 14).

With regard to radiotherapy, concerns have historically been raised about increased radiosensitivity and the potential risk of radiation-induced malignancies in BRCA mutation carriers. However, current clinical evidence does not support a higher incidence of severe toxicity or contraindicate its use. On the contrary, radiotherapy remains an integral component of BCT and has been shown to significantly reduce IBTR, even in BRCA-mutated patients (13, 17). From a biological standpoint, BRCA-deficient tumor cells may exhibit increased radiosensitivity due to impaired DNA repair capacity. However, clinical studies have not consistently demonstrated differences in radiotherapy outcomes or toxicity between BRCA mutation carriers and non-carriers, suggesting that this biological susceptibility does not directly translate into clinically significant differences (33).

These findings regarding loco-regional and systemic treatments support a personalized treatment approach in BRCA mutation carriers, where therapeutic decisions are guided not only by tumor stage and phenotype but also by underlying genetic susceptibility.

## 8 Patient-centered outcomes

Quality of life (QoL) is a key component of surgical decision-making in BRCA mutation carriers, given the comparable survival outcomes of BCT and mastectomy. Several studies have consistently demonstrated that BCT is associated with better patient-reported outcomes in terms of body image, sexual functioning, and overall psychosocial well-being compared with mastectomy, particularly when reconstruction is not performed (34–36). Preservation of the breast has been shown to positively influence self-perception and femininity, which are especially relevant in younger BRCA carriers who are often diagnosed at an earlier age than sporadic cases (14).

Conversely, mastectomy—especially bilateral risk-reducing mastectomy—has been associated with increased physical and psychological burden in the short term, including impaired body image, reduced sexual satisfaction, and higher rates of surgical complications, although these effects may improve over time (36, 37). The use of immediate breast reconstruction can mitigate some of these negative outcomes, but does not always fully restore preoperative levels of QoL, particularly in terms of sensory changes and body image perception (37, 38).

Importantly, BRCA mutation carriers face unique psychosocial challenges that may influence QoL independently of surgical choice. The awareness of an increased contralateral breast cancer risk often leads to heightened anxiety and decisional conflict, which can drive patients toward more radical surgical options such as bilateral mastectomy despite the absence of a clear survival benefit (14, 39). In this context, some studies have reported lower cancer-related anxiety and greater perceived peace of mind following mastectomy, suggesting that the psychological impact of risk reduction may offset the negative physical consequences of more extensive surgery (40). Comparative studies between BCT and mastectomy in BRCA carriers indicate that overall QoL outcomes tend to converge over time, with no consistent long-term differences in global health status or satisfaction with care, although specific domains such as body image and sexual functioning generally remain more favorable after BCT (41). Furthermore, patient satisfaction appears to be strongly influenced by preoperative counseling and the degree to which surgical decisions align with individual preferences and expectations, rather than by the surgical procedure itself (42).

Overall, in BRCA mutation carriers, QoL outcomes are therefore shaped by a complex interplay between physical sequelae, psychological factors, and perceived risk reduction, underscoring the need for comprehensive counseling and shared decision-making when selecting the optimal surgical approach.

## 9 Current guidelines

Current international guidelines broadly agree that both BCT and mastectomy are acceptable surgical options for women with BRCA1/2-associated breast cancer. Major organizations such as the National Comprehensive Cancer Network, European Society for Medical Oncology, and American Society of Clinical Oncology recommend that BRCA mutation status alone should not preclude BCT, provided that standard oncologic criteria are met, including the ability to achieve negative margins and deliver adjuvant radiotherapy (43–45). These recommendations are supported by a growing body of evidence showing comparable survival outcomes between BCT and mastectomy in BRCA mutation carriers (13, 24).

At the same time, current guidelines consistently emphasize the increased lifetime risk of contralateral breast cancer in BRCA carriers and recommend discussing risk-reducing strategies, including contralateral prophylactic mastectomy or bilateral mastectomy in selected high-risk patients (3, 45). However, prophylactic surgery is not mandatory and should be individualized based on age, tumor characteristics, family history, and patient preferences. All major guidelines highlight the importance of multidisciplinary evaluation and preoperative genetic counseling to support shared decision-making and optimize long-term outcomes (43, 44). The importance of a multidisciplinary team approach in guiding treatment decisions has been further emphasized in recent literature, particularly in complex scenarios (46).

Overall, contemporary recommendations endorse a personalized surgical approach in BRCA-associated breast cancer, balancing oncologic safety, risk reduction, and QoL, rather than favoring a single standard surgical strategy for all patients.

## 10 Controversies

Despite the growing body of evidence supporting the oncologic safety of BCT in BRCA mutation carriers, several important controversies remain, largely related to the limitations of the available studies. Most of the current evidence is derived from retrospective observational cohorts, often with heterogeneous populations, long inclusion periods, and variability in systemic therapies and radiotherapy techniques over time, which may significantly influence outcomes (13, 24). In addition, selection bias is a major concern, as patients undergoing BCT are frequently younger, have smaller tumors, and receive more aggressive adjuvant treatments compared with those undergoing mastectomy.

Another key limitation is the difficulty in distinguishing true local recurrences from new primary tumors, particularly in BRCA carriers, where the risk of second primaries is intrinsically elevated. Although survival outcomes appear comparable between BCT and mastectomy, the consistently higher incidence of ipsilateral breast events observed after BCT, particularly with longer follow-up, remains a relevant clinical concern. Furthermore, follow-up duration varies considerably across studies, and shorter follow-up may underestimate late ipsilateral events, which are known to occur more frequently in this population. Therefore, the oncologic safety of BCT should be interpreted within the context of this increased local risk and discussed carefully during shared decision-making.

Importantly, no randomized controlled trials directly comparing BCT and mastectomy in BRCA mutation carriers are available, largely due to ethical and practical constraints, leaving clinicians to rely on lower levels of evidence. Additional limitations include incomplete adjustment for confounding factors such as oophorectomy, use of PARP inhibitors, and evolving systemic therapies, all of which may impact recurrence risk and survival. Consequently, while current data suggest that BCT is a reasonable option in selected patients, these methodological constraints continue to fuel debate and highlight the need for more robust prospective and contemporary data.

Outcomes reported in historical cohorts may not accurately reflect the prognosis of contemporary BRCA mutation carriers treated in the era of PARP inhibitors, modern radiotherapy, and tailored systemic treatments. Although findings across observational studies and meta-analyses are largely consistent, the current evidence should be interpreted with caution, as definitive conclusions regarding the comparative effectiveness of BCT and mastectomy remain limited by the lack of prospective randomized data.

## 11 Future perspectives

Future perspectives in the management of BRCA-associated breast cancer are increasingly centered on personalized, biology-driven treatment strategies that integrate genetic, molecular, and clinical factors to optimize outcomes. The expanding understanding of homologous recombination deficiency is likely to refine patient selection not only for systemic therapies but also for local treatment decisions, potentially identifying subgroups in which BCT can be safely pursued with minimal risk (30, 31). In this context, genomic profiling and biomarkers of DNA repair capacity may help stratify patients according to recurrence risk and guide surgical planning beyond BRCA status alone.

An important unmet need in this field is the availability of prospective contemporary data specifically addressing surgical outcomes in BRCA mutation carriers. Most of the current evidence supporting BCT is derived from retrospective studies with heterogeneous populations, treatment strategies, and follow-up durations. Future prospective multicenter studies and dedicated registries should therefore evaluate not only traditional oncologic endpoints such as local recurrence, disease-free survival, and overall survival, but also patient-reported outcomes, quality of life, decisional regret, and long-term surveillance burden. Such studies may provide a more accurate estimate of the true oncologic impact of breast conservation in the era of modern systemic therapies and advanced imaging.

Another promising area of investigation is the development of biomarker-guided surgical selection models. While BRCA status currently represents the main genetic determinant influencing surgical decision-making, it is increasingly evident that considerable biological heterogeneity exists among BRCA-associated tumors. Future research should explore the integration of biomarkers related to homologous recombination deficiency, genomic instability, treatment response, and minimal residual disease with conventional clinicopathological factors. In particular, the degree of response to neoadjuvant therapy, achievement of pathological complete response, and molecular signatures associated with recurrence risk may help identify patients who are optimal candidates for breast conservation despite carrying a germline BRCA mutation.

The growing use of PARP inhibitors in both early-stage and metastatic settings represents a major step toward precision oncology, with trials demonstrating improved DFS in high-risk BRCA carriers (31). Their integration into neoadjuvant and adjuvant treatment strategies may further reduce local and distant recurrence rates, potentially influencing the balance between BCT and mastectomy in favor of more conservative approaches. Similarly, advances in systemic therapy, including immunotherapy for triple-negative breast cancer, may contribute to improved tumor control and expand the eligibility for breast conservation (47, 48). Although these therapeutic advances provide a strong biological and clinical rationale for expanding the use of breast-conserving therapy in selected BRCA mutation carriers, direct evidence demonstrating that they specifically improve the oncologic safety of BCT in this population remains limited. Therefore, this should be regarded as a promising and evolving perspective that warrants validation in prospective contemporary studies.

Another relevant future direction concerns the development of comprehensive risk prediction tools capable of combining germline mutations, tumor biology, imaging findings, treatment response, and patient-specific characteristics. Such models could provide individualized estimates of ipsilateral and contralateral breast cancer risk, thereby supporting shared decision-making and helping clinicians tailor the extent of surgery according to the patient’s actual risk profile rather than relying on mutation status alone. Ultimately, this approach could facilitate a transition from a mutation-centered strategy to a truly precision-based surgical management paradigm.

## 12 Conclusions

Both cohort studies and meta-analyses consistently demonstrate that, although BCT is associated with a higher risk of IBTR, this does not translate into inferior OS, breast cancer–specific survival, or distant recurrence outcomes (13, 24, 27). This apparent discrepancy may be explained by the underlying tumor biology in BRCA carriers, characterized by impaired DNA repair mechanisms and increased responsiveness to systemic therapies.

Advances in systemic therapies, including platinum-based chemotherapy and PARP inhibitors, together with improvements in radiotherapy and imaging, have further contributed to enhanced local and systemic disease control, thereby increasing the feasibility of breast-conserving approaches in this high-risk population.

Importantly, surgical decision-making in BRCA mutation carriers should not rely solely on oncologic outcomes but must also incorporate patient-centered considerations, including QoL, body image, and psychological well-being. While BCT is generally associated with better cosmetic and psychosocial outcomes, mastectomy may provide greater reassurance in terms of risk reduction, highlighting the importance of individualized, shared decision-making. Nevertheless, significant limitations persist in the available evidence, including the absence of randomized controlled trials, heterogeneity among studies, and evolving treatment paradigms, which continue to fuel ongoing debate. In this context, BRCA-associated breast cancer should be viewed not only as a high-risk condition but also as a biologically distinct entity with specific therapeutic vulnerabilities. Future strategies should focus on personalized treatment approaches integrating genetic, molecular, and clinical factors to optimize both oncologic outcomes and QoL.

Overall, the current body of evidence from cohort studies focusing on the treatment of BRCA-associated breast cancer suggests that, although BCT may be associated with a slightly increased risk of local breast events, it does not adversely impact survival outcomes and can be considered a valid alternative to mastectomy in appropriately selected BRCA mutation carriers. However, this approach should be accompanied by careful patient selection, individualized multidisciplinary counseling, shared decision-making, and structured long-term surveillance, with particular attention to the ongoing risks of ipsilateral and contralateral breast cancer. In the context of contemporary multimodal treatment strategies, BCT represents a reasonable option for selected patients, while acknowledging that further prospective contemporary studies are needed to better define its long-term oncologic outcomes.
